# Plant Species Composition Alters the Sign and Strength of an Emergent Multi-Predator Effect by Modifying Predator Foraging Behaviour

**DOI:** 10.1371/journal.pone.0070258

**Published:** 2013-08-01

**Authors:** Andrew Wilby, Linda Anderson Anglin, Christopher M. Nesbit

**Affiliations:** 1 Lancaster Environment Centre, University of Lancaster, Lancaster, Lancashire, United Kingdom; 2 School of Agriculture, Policy and Development, University of Reading, Reading, Berkshire, United Kingdom; University of California, Berkeley, United States of America

## Abstract

The prediction of pest-control functioning by multi-predator communities is hindered by the non-additive nature of species functioning. Such non-additivity, commonly termed an *emergent multi-predator effect*, is known to be affected by elements of the ecological context, such as the structure and composition of vegetation, in addition to the traits of the predators themselves. Here we report mesocosm experiments designed to test the influence of plant density and species composition (wheat monoculture or wheat and faba bean polyculture) on the emergence of multi-predator effects between *Adalia bipunctata* and *Chrysoperla carnea,* in their suppression of populations of the aphid *Metopolophium dirhodum.* The mesocosm experiments were followed by a series of behavioural observations designed to identify how interactions among predators are modified by plant species composition and whether these effects are consistent with the observed influence of plant species composition on aphid population suppression. Although plant density was shown to have no influence on the multi-predator effect on aphid population growth, plant composition had a marked effect. In wheat monoculture, *Adalia* and *Chrysoperla* mixed treatments caused greater suppression of *M. dirhodum* populations than expected. However this positive emergent effect was reversed to a negative multi-predator effect in wheat and faba bean polyculture. The behavioural observations revealed that although dominant individuals did not respond to the presence of faba bean plants, the behaviour of sub-dominants was affected markedly, consistent with their foraging for extra-floral nectar produced by the faba bean. This interaction between plant composition and predator community composition on the foraging behaviour of sub-dominants is thought to underlie the observed effect of plant composition on the multi-predator effect. Thus, the emergence of multi-predator effects is shown to be strongly influenced by plant species composition, mediated, in this case, by the provision of extra-floral nectar by one of the plant species.

## Introduction

In recent years, there has been considerable interest in the relationship between community attributes and the provision of ecosystem services, such as pest population suppression. Although numerous studies have focussed on the role of natural enemy species richness or composition in determining prey population suppression, predicting the impact of natural enemy community change on ecosystem functioning is often hindered by emergent multi-predator effects; the functional impact of a species assemblage is not a straight-forward linear combination of the constituent species impacts [1]. As multi-predator communities are the norm in real ecosystems, understanding the ecological mechanisms underlying multi-predator effects is fundamental to our understanding of prey population regulation. This understanding is required both for the effective management of biological control [2], and for the identification of conflicts between biological control and conservation of biodiversity in agricultural systems [3], [4].

Emergent multi-predator effects on prey population suppression are classically thought to arise when there is a trait-mediated or density-mediated interaction [5] between predator species that modifies their combined impact on the prey species. For example, predator species can facilitate each other by inducing behavioural changes in the prey or in the predators themselves [6], [7], resulting in positive emergent effects on predation rate (prey risk enhancement). Interactions between species can also result in negative multi-predator effects (prey risk reduction) if, for example, interference between heterospecific individuals reduces predation rate. Moreover, intraguild predation, which has been shown to be extremely widespread in natural communities [8], may also result in reduced prey suppression [9].

Aside from interactions among species, general biodiversity – ecosystem functioning theory tells us that emergent multi-species effects also arise when there is niche-differentiation between species, such that multispecies communities occupy a greater proportion of total niche space [10]. Under this scenario, increasing the number of predator species, while fixing predator density, results in a positive emergent multi-predator effect only if the predation rate of prey is limited by intraspecific competition [11], [12], because replacing some individuals of one predator species with another increases the proportion of the prey population that is susceptible to predation and relaxes competition between individual predators [13].

It is clear that multi-predator assemblages can exhibit a range of emergent multi-predator effects depending on the relative strengths of a range of potential mechanisms. There is also evidence that the same multi-predator assemblage can exhibit different emergent effects in different environmental contexts; multi-predator effects are not determined solely by fixed traits of the predator species concerned, but vary depending on how the environment affects the underlying mechanisms. For example, prey life-history [14], [15], [16] can facilitate niche differentiation leading to increased functional complementarity among predators, whereas increased habitat structural complexity has been shown to reduce the impact of intraguild predation by reducing encounter rates and providing refuges for intraguild prey [17], [18], [19]. It has also been suggested that negative emergent multi-predator effects may be more likely to occur on structurally simple plants, such as grasses, compared with more complex species, but this has yet to be formally tested [20]. Finally, theoretical prediction and recent experimental evidence suggests that food availability may affect the emergence of multi-predator effects. For example increased prey diversity may promote functional complementarity among predator species by allowing differentiation among predators in their use of prey, or by changing predator behaviour such that facilitative interactions among predators are modified [12].

Here we conduct experimental tests to determine whether plant density and plant composition (monoculture vs biculture) affect the emergent multi-predator effects occurring between two aphidophagous predators. Our plant species include one plant that produces extra-floral nectar as a supplementary/alternative food source, which is predicted to affect foraging activity. We formally test the hypotheses: 1) that increased plant density reduces the influence of negative intraguild interactions leading to more positive emergent effects on prey suppression; 2) that the presence of extra-floral nectar provides an alternative food source leading to reduced prey consumption; and 3) that foraging responses of predators to changing resource environments result in modification of emergent multi-predator effects.

## Methods

A combination of mesocosm experiments and independent behavioural observations were undertaken to test the effect of plant density and species composition on the emergence of multi-predator effects, and to identify the behavioural mechanisms underlying these effects. The experimental system included two commonly co-occurring aphidophagous predators, adult *Adalia bipunctata* (Coccinellidae) and second instar larvae of *Chrysoperla carnea* (Chrysopidae), and a common prey species, the cereal feeding aphid *Metopolophium dirhodum.* This combination of predator life stages was chosen to generate an asymmetric negative interspecific interaction in the multi-predator treatment, with the larger *Adalia* adults being the potential intraguild predator and dominant competitor of *Chrysoperla* larvae. This combination facilitated a direct test of the hypothesised moderating effect of plant density on negative intraguild interactions.

### Mesocosm Experiment

Experimental mesocosms (60×60×60 cm; Bugdorm2, MegaView Science Co. Ltd, Taiwan) housed in an unheated glasshouse were used to measure the impact of adult *Adalia bipunctata* and second instar larvae of *Chrysoperla carnea* on population growth of *M. dirhodum*. Four predator treatments were used in the experiment: *Adalia* alone, *Chrysoperla* alone, the two species in combination, and a predator-free control. A substitutive design was used, such that four individual predators were introduced into each cage, with two of each species in the mixed predator treatment. These predator treatments were fully crossed in a factorial combination with three vegetation treatments comprising low-density wheat monoculture (*Triticum aestivum* var Tybalt), high-density wheat monoculture, and a mixed culture of wheat and faba bean (*Vicia faba* var Hobbit). Plants were sown in compost in seed trays (35×21×6.5 cm) separated into 5 strips (each 6.5×21×6.5 cm). The low-density wheat treatment had three plants in each of three strips, the high-density wheat had three plants in each of five strips, and the wheat/bean polyculture had three wheat plants in three strips plus three bean plants in two strips. All plants were reared from seed in a glasshouse in compost (John Innes #2 compost) and were approximately five weeks old at the start of the experiment. Faba bean was used as the second species because it is commonly inter-cropped with cereals and because it produces extra-floral nectar and is, therefore, a source of supplementary/alternative resources for predators.

At the start of each experimental run, 30 third or fourth instar *M. dirhodum* (in three groups of ten) were introduced into each cage. *M. dirhodum* were obtained from a single-clone culture housed at Lancaster University. After 24 h the appropriate number of predators was introduced to each cage. All predators were purchased immediately prior to each experimental run (Fargro Ltd, UK) and were maintained on an aphid-free diet (buckwheat seeds) at 5°C for approximately 24 h before use. 48 h after introduction of the predators, each cage was destructively harvested and the number of surviving *M. dirhodum* counted. For logistical reasons, the experiment was replicated across seven temporal blocks, with each block comprising the full set of treatment combinations.

The per capita impact of predators on the population growth of *M. dirhodum* was calculated as *m = ln(N_c,t_/N_p,t_)/4*, where *N_p,t_* is the aphid population size at harvest time *t* under the predator treatment *p*, and *N_c,t_* is the mean aphid population size at harvest time *t* across the three control cages (those without predators) in the same block. The variable *m* represents the extent to which the intrinsic population growth rate *r* is reduced by each predator assuming *dN/dt = rN* [21]. In order to calculate the multi-predator effect on *m* values, expected prey population suppression was calculated as the average of the *m* values for the constituent single species treatments under the same vegetation treatment in the same block. The emergent multi-predator effect (MPE) was defined as the deviation from expectation: = *ln(O_m_/E_m_)*, where *O_m_* is the observed *m* value and *E_m_* is the expected *m* value for the mixed predator treatment. Values >0 indicate prey suppression significantly higher than expected (risk enhancement), values <0 indicate lower prey suppression than expected (risk reduction).

Analysis of per capita impact and MPE were done by ANOVA in R [22]. The significance of factors was assessed by deletion from the full model. The statistical significance of terms was assessed by F–tests of the larger and reduced model at each deletion [23]. Model simplification also tested the effect of replacing the factor *plant* with a contrast representing the effect of plant richness (high and low-density wheat treatments vs. wheat/bean mix) followed by a contrast testing the effect of wheat density [23]. Differences between factor levels were also tested using Tukey’s HSD test [23].

### Behavioural Observations

In order to provide mechanistic explanations for the population responses observed in the mesocosm experiments, a series of observations of predator interactions were undertaken in an experimental arena (30×30×30 cm Bugdorm1, MegaView Science Co. Ltd, Taiwan), modified to have one transparent side to facilitate observation. The experiment was designed to test the effect of interpredator interactions (conspecific and heterospecific) on foraging behaviour and how these were modified by plant composition. Two plant pots (7-cm diameter), each containing a single plant, were placed in the arena in one of two arrangements: two wheat (*Triticum aestivum* var Tybalt) or one wheat and one faba bean (*Vicia faba* var Hobbit). In all cases, ten individual *M. dirhodum* were placed on an upper leaf of the wheat plant in wheat/bean combinations, or on one of the pair of wheat plants in the wheat/wheat combinations. *M. dirhodum* all came from a single-clone culture maintained at Lancaster University. Predator foraging observations were made of *Adalia* adults and second-instar *Chrysoperla* larvae alone, in combination with a conspecific, and in combination with a heterospecific individual. Approximately eight independent trials of each predator combination were made in each of the plant composition treatments. All predators were purchased from a biological control supplier (Fargro Ltd, UK) prior to the experiments and were maintained on an aphid-free diet at 5°C before use. All observations were made in a controlled-environment room at 20°C and all predators were allowed to acclimatise for 1 h prior to observations. At the start of each observation period (approx. 30 min), a clean transparent bridge (7×3 cm) was positioned to join the plant pots such that the each end of the bridge was touching the stem of the plant. The appropriate predator combination was then released at the centre point on the bridge (equidistant from the two plants) and the following data were recorded for each predator: total time spent on each plant; time spent on the bridge, soil surface or other areas in the cage; time spent feeding on aphids; and time spent feeding at extra-floral nectaries (wheat/bean treatment only).

Analysis of predator foraging was done from two different perspectives. First, in all treatments that received two predator individuals, the percentage of total foraging time (of both predators) that was spent on the infested or alternative plant was analysed. This gives an indication of how foraging pressure on the two plants was influenced by the predator treatments and the plant treatments. Second, the foraging behaviour of individual predators was analysed. In predator treatments with two conspecific individuals, the individual spending most time on the aphid-infested plant was deemed to be the dominant and the individual spending least time on the aphid-infested plant the sub-dominant. This revealed whether similar dominance hierarchies existed within and between species, and whether these were affected by the identity of the alternative (aphid-free) plant offered to the predators. As with the mesocosm experiment, all analyses were performed by ANOVA in R [22]. The significance of factors was assessed by deletion from the full model and the statistical significance of terms was assessed by F–tests of the larger and reduced model at each deletion. Where necessary, differences between factor levels were tested using Tukey’s HSD test [23]. We report untransformed percentage data as transformation was not required to avoid mis-specification of models. Analyses were repeated with arcsine transformed data producing qualitatively similar results that are not presented here.

## Results

### Mesocosm Experiment

**Figure 1:**
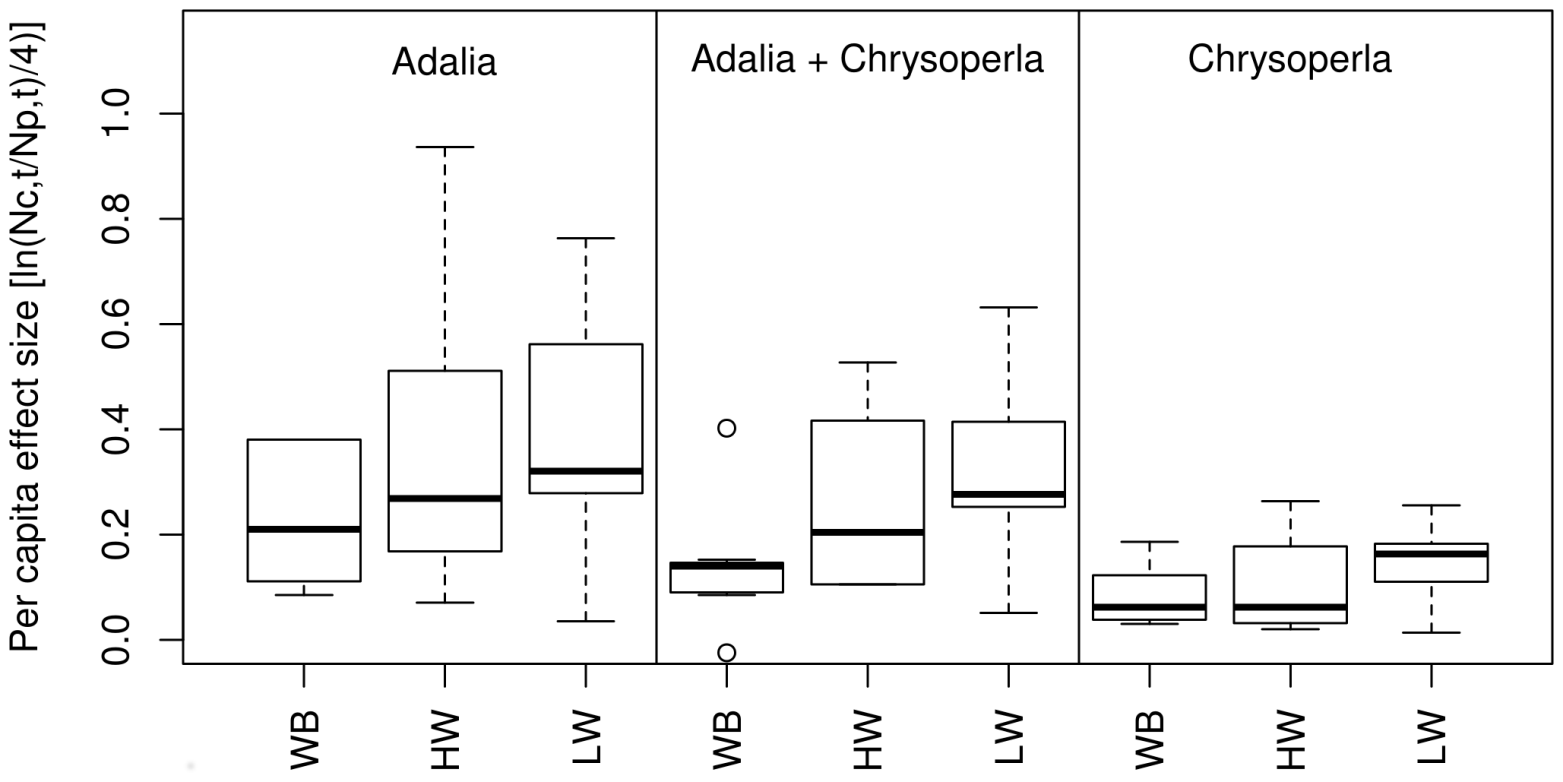
Suppression of aphid population growth rate in *Adalia*, *Chrysoperla* and mixed predator treatments across the plant treatments: wheat plus bean (WB), high-density wheat (HW) and low-density wheat (LW). Bars and boxes denote medians and IQR. Dashed lines extend to minimum and maximum values, open circles denote outliers.

Per capita suppression of aphid population growth (*m*) varied significantly between both the predator treatments (F_2,52_ = 14.00; P<0.001) and plant treatments (F_2,50_ = 4.55; P = 0.015; Fig. 1) and among blocks (F_6,50_ = 5.73; P<0.001), but there was no significant interaction between plant and predator treatments. Suppression of the prey population was lowest in the wheat and bean polyculture treatment, significantly lower than in the low-density wheat treatment (adjusted P = 0.014, Tukey HSD) and marginally lower than the high-density wheat treatment (adjusted P = 0.10, Tukey HSD). The contrast comparing aphid population growth suppression (*m*) in treatments with both wheat and bean plants compared with the wheat-only treatments was significant (coefficient = 0.11; F_1,56_ = 6.94; P = 0.011; Fig. 1) showing that suppression of aphid population growth was higher in the wheat monocultures compared with the wheat and bean polyculture treatment. There was no significant difference in aphid population suppression between the high and low density wheat treatments.

All predator treatments caused significant suppression of aphid population growth but suppression was highest in the *Adalia* treatment (Fig. 1). The *Chrysoperla* treatment caused significantly lower suppression than the *Adalia* plus *Chrysoperla* treatment (coefficient = −0.13; adjusted P = 0.013, Tukey HSD) or the *Adalia* treatment (coefficient = −0.23; adjusted P<0.001, Tukey HSD). The *Adalia* treatment did not result in significantly greater suppression than the *Adalia* plus *Chrysoperla* treatment (adjusted P = 0.078, Tukey HSD).

**Figure 2:**
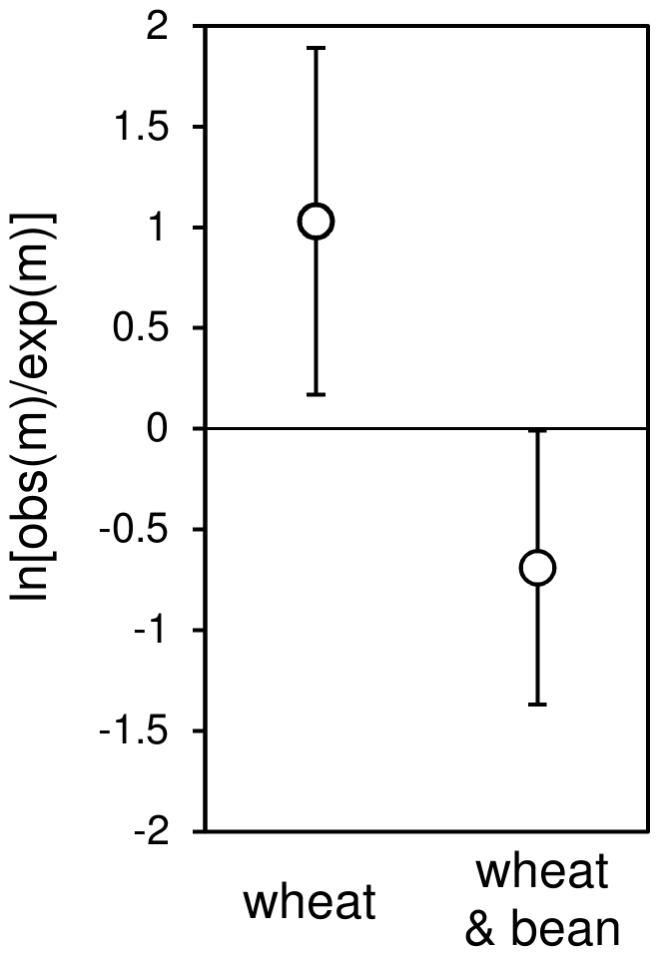
The observed emergent multi-predator effect on *M.* *dirhodum* population suppression by mixed assembages of *C. carnea* and *A. bipunctata* in mesocosms containing wheat monoculture, and wheat and bean polyculture. Values >0 indicate higher prey suppression than expected, <0 indicate lower prey suppression than expected. Error bars denote ± one standard error of the mean.

Analysis of MPE revealed a significantly lower MPE where both wheat and bean were present compared with the wheat monoculture treatments (F_1,16_ = 6.06; P = 0.026). This analysis also revealed that there was a change in the nature of the MPE from significantly positive in the wheat treatments (greater than expected prey suppression) to negative (lower than expected prey suppression) in the wheat plus faba bean polyculture treatment (Fig. 2).

### Behavioural Observations

**Figure 3:**
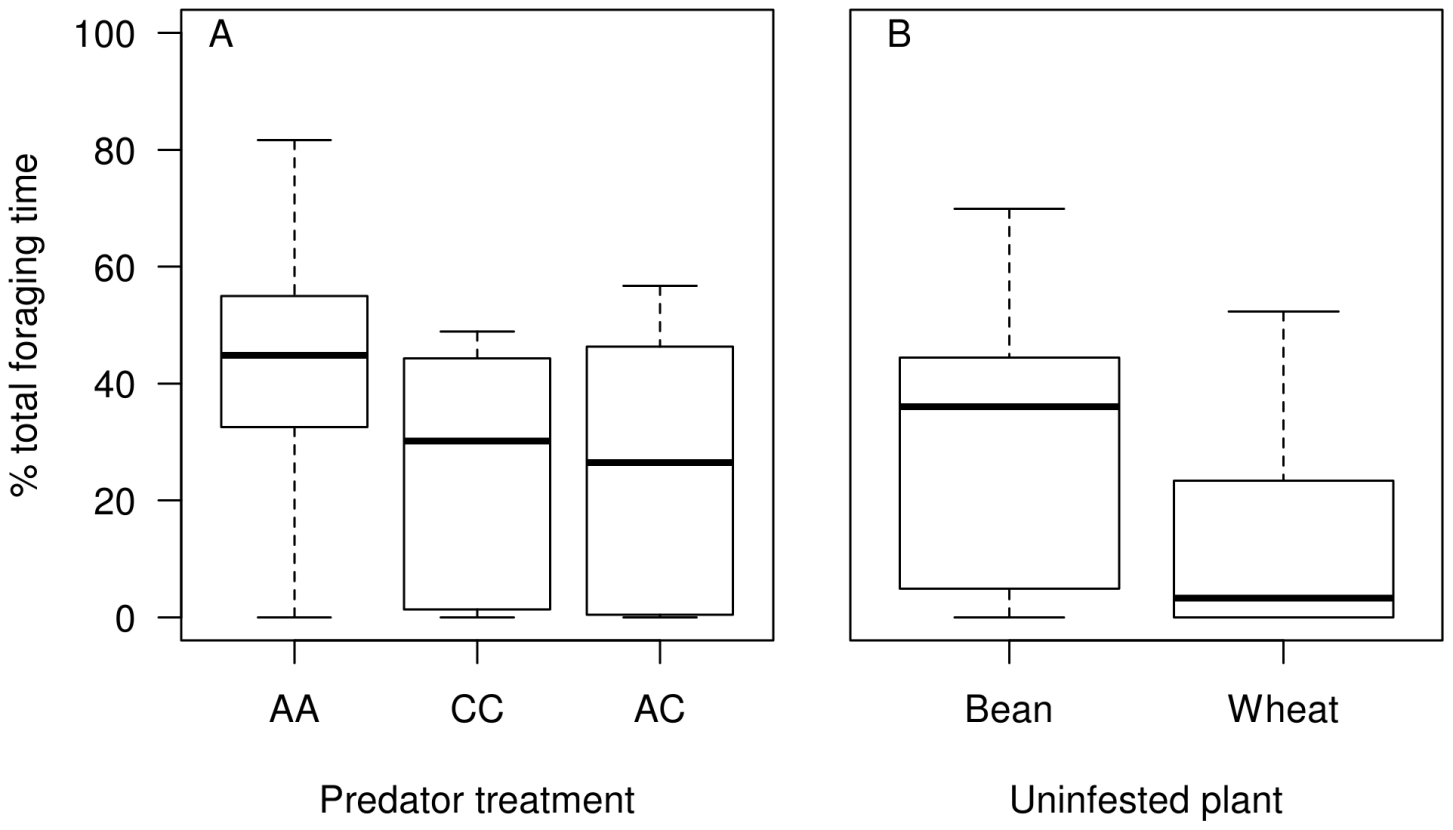
Total foraging time (%) allocated: (a) by predators to the aphid-infested wheat plant under different predator treatments; and (b) to the aphid-free plant in the different plant identity treatments. Predator treatments are labelled: AA - two *Adalia* individuals; CC - two *Chrysoperla* individuals; and AC - one *Adalia* plus one *Chrysoperla* individual. Bars and boxes denote medians and IQR. Dashed lines extend to minimum and maximum values, open circles denote outliers.

The percentage of total observation time spent foraging on the plant infested with aphids was significantly affected by predator treatment (F_2,45_ = 3.93; P = 0.027), but not by plant treatment, and there was no significant interaction between plant and predator treatments. The predator effect arose from significantly less foraging time on the aphid-infested plant in the *Adalia* plus *Chrysoperla* combination treatment than in the *Adalia* treatment (coefficient −19.4; adjusted P = 0.042, Tukey HSD; Fig. 3a), other comparisons were not significant. By contrast, the percentage of time foraging on the alternative aphid-free plant was significantly affected by the identity of the plant, with significantly less foraging time allocated when the alternative aphid-free plant was wheat compared with when it was bean (Coefficient = −15.92, F_1,46_ = 8.22; P = 0.006; Fig. 3b). The amount of time spent foraging away from the plants (on the cage, bridge or soil surface) was significantly higher when the alternative plant was wheat compared with when it was bean (Coefficient = 17.28, F_1,46_ = 6.00.; P = 0.018).

**Figure 4:**
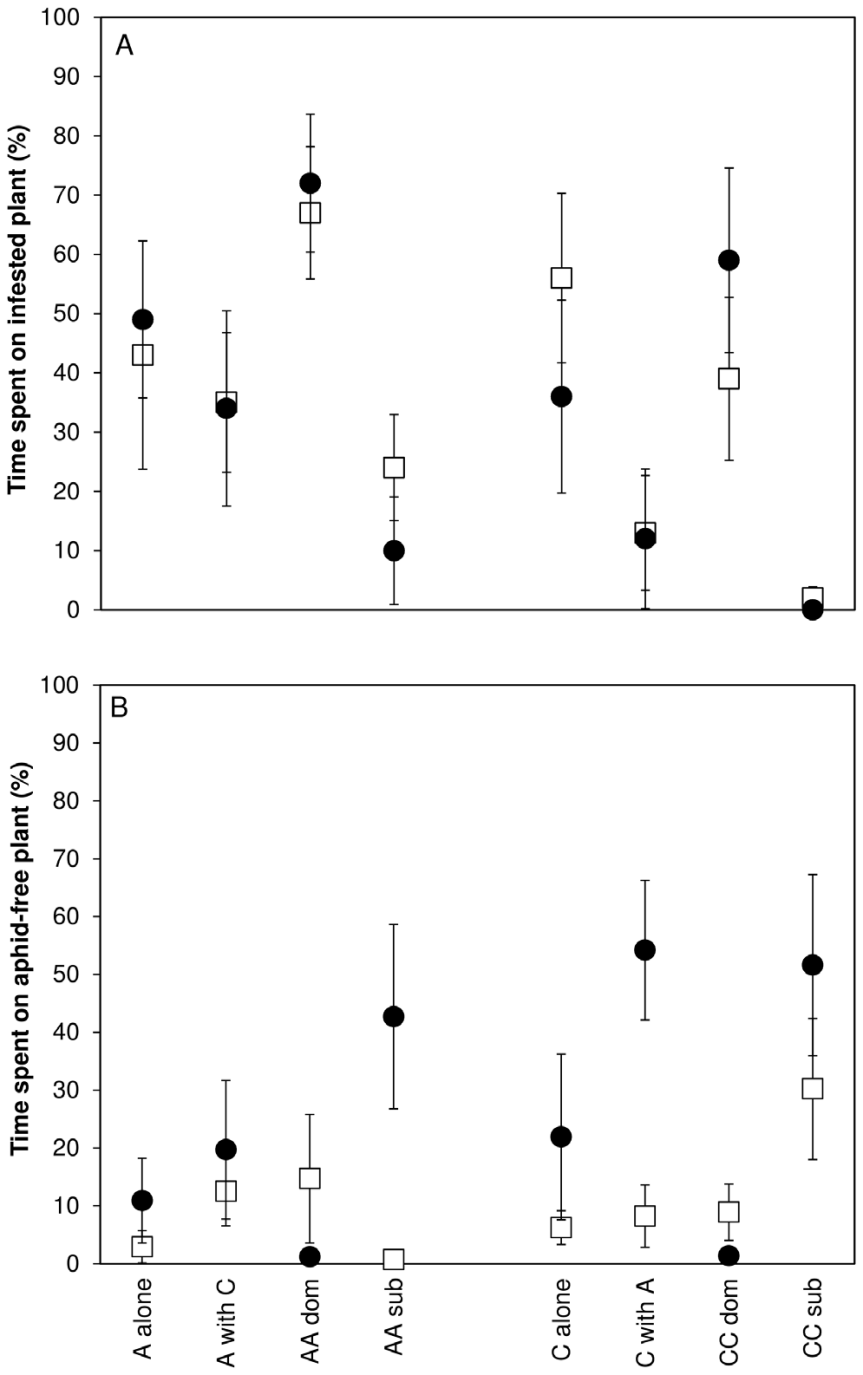
Time allocation of individual predators to: (a) the aphid-infested plant; and (b) to the aphid-free plant, in wheat (open squares) and wheat plus bean (filled circles) treatments. A denotes *Adalia*, *C* denotes *Chrysoperla*, AA dom and CC dom denote the dominant individual in the two-individual *Adalia* and two-individual *Chrysoperla* treatments respectively, whereas AA sub and CC sub denote the sub-dominant individual in these treatments. Error bars denote ± one standard error of the mean.

The time budgets of individual insects showed that *Adalia* spent a larger percentage of time foraging on the aphid-infested plant than *Chysoperla* (41% vs 25%; F_1,118_ = 5.08; P = 0.026) and there was a highly significant effect of the predator combination within species (F_6,112_ = 7.74; P<0.001; Fig. 4). In all trials with two individuals, significant dominance was shown. When *Adalia* was paired with a conspecific individual, the dominant individual spent significantly greater time on the aphid-infested plant than the sub-dominant individual (adjusted P<0.001, Tukey HSD; Fig. 4a). Similarly, dominant *Chrysoperla* individuals spent significantly greater time on the aphid-infested plant than the sub-dominant conspecific individuals (adjusted P = 0.002, Tukey HSD). When heterospecific individuals were paired, *Adalia* tended to be dominant, spending a greater proportion of time foraging on the aphid-infested plant than *Chrysoperla* (adjusted P<0.001, Tukey HSD).

Time spent foraging on the alternative aphid-free plant was significantly affected by whether the alternative was wheat or bean (F_1, 104_ = 9.79; P = 0.002; Fig. 4b) and by the predator treatment (F_7, 104_ = 3.55; P = 0.002), and these two factors had a statistically significant interaction (F_7, 104_ = 2.58; P = 0.017). Generally, more time was spent by predators on bean plants than aphid-free wheat plants (26% and 11% respectively; adjusted P = 0.002, Tukey HSD), and the plant species identity had a significant impact on foraging time allocated to the aphid-free plant by sub-dominant individuals in conspecific and heterospecific pairs (Fig. 4b).

Feeding at the extra-floral nectaries on the bean plant was recorded in 10 *Chrysoperla* individuals and 10 *Adalia* individuals out of the 24 individuals of each species observed in wheat/bean trials. On average the *Adalia* individuals that visited nectaries spent 25.1% (±6.7 s.e.) of their foraging time at a nectary compared with 21.3% (±3.5 s.e.) for *Chrysoperla*. Of the 24 trials in which two predators were present, only in three trials were both predators observed to feed on nectar (one conspecific *Adalia* trial and two heterospecifc trials).

## Discussion

The data reported here show that plant composition, but not plant density, can have a strong impact on the sign and strength of emergent multi-predator effects on prey suppression. Previous studies of the effect of vegetation on the functioning of multi-predator communities have shown variable results, leading to the suggestion that structural complexity of vegetation or habitat may be more important than plant identity in determining the outcome and magnitude of emergent multiple predator effects [24]. It is likely, therefore, that both habitat complexity and plant identity can modify multi-predator effects depending on the nature of habitat and plant identity differences and on the response of the predator species to these differences.

Several studies have shown an effect of habitat complexity on emergent multi-predator effects caused, for example, by lower rates of intraguild predation in more complex environments [17], [25], though the opposite has also been found with habitat complexity increasing intraguild predation [26]. Increasing habitat complexity has also been shown to change the outcome of multi-predator effects by preventing facilitation among predators [27]. In the experiment reported here, there was no significant effect of plant density on prey suppression or on the emergent multi-predator effect, suggesting that plant density did not interfere with prey consumption or affect interactions between the predator species sufficiently to impact significantly on prey consumption. However, plant density is only one component of habitat complexity, and our results support earlier studies which have shown no effect of plant density, but strong effects of plant morphological complexity on prey consumption, due to the greater opportunity for prey refuge that more complex morphologies provide [26].

Although the experiments revealed little effect of plant density on multi-predator effects, they did reveal strong effects of plant composition on emergent multi-predator effects, characterised by a switch from positive (increased prey suppression) multi-predator effects in wheat monocultures to negative (deceased prey suppression) multi-predator effects in wheat and bean polyculture. Although some previous experiments have failed to show an effect of plant identity on diversity effects among aphidophagous predators [28], a recent study showed that the presence of a waxy plant surface structure can negate predator richness effects on prey consumption by changing foraging behaviour such that facilitative interactions among predators are compromised [29]. Clearly, if plant identity changes modify predator behaviour, or the response of prey to predators, we may expect multi-predator effects to be altered.

Under a substitutive experimental design, emergent multi-predator effects reflect a difference in impact of interspecific interactions compared with intraspecific interactions on prey suppression [30]. Therefore, assuming that similar interactions occurred between predator individuals in the observation cages and the mesocosms, it would be expected that the observations would reveal differences in foraging behaviour between conspecific and heterospecific pairings, and that these differences would vary depending on the identity of the plants. Although we did not observe any instances of intraguild predation or cannibalism, our observations showed that both heterospecific and conspecific parings exhibited strong dominance, with one individual spending a higher proportion of time on the aphid-infested plant. They also revealed that the identity of the aphid-free plant made little difference to the time allocation of individual predators to the aphid-infested plant. However, consistent with the records of nectar feeding by both predator species, foraging allocation to the aphid-free plant was markedly higher, and time spent off the plants markedly lower, in the wheat/bean treatment compared with the wheat/wheat treatment. Analysis of the behaviour of individual foragers showed that this change in foraging allocation was due to changing behaviour of sub-dominant individuals in response to the species identity of the aphid-free plant. Foraging on the aphid-free plant by sub-dominant *Adalia* individuals in the conspecific pairings, and *Chysoperla* individuals in heterospecific pairings, increased markedly when bean was present compared with wheat. There was no such effect with sub-dominant *Chrysoperla* in conspecific pairings. We propose that the interactive effect of predator and plant treatments on the foraging behaviour of sub-dominant *Chrysoperla* individuals may underlie the observed impact of plant identity on the multi-predator effect in the mesocosm experiment.

Why should changes in the behaviour of sub-dominant individuals, which generally did not forage on the aphid infested plant, affect the multi-predator effect on prey suppression? Positive multi-predator effects can arise from resource-use complementarity among the predators, such that a greater proportion of the aphid population is susceptible to predation, or from facilitation among predators. For example, facilitation between foliar and ground foraging aphidophagous predators has been commonly reported [6], [31]. Facilitation arises because many aphid species, including *M. dirhodum* [32], employ anti-predator dropping behaviour in response to foliar predators, which makes them more susceptible to predation by ground foraging predators. Although our foraging observations revealed little impact of plant identity on the intensity of foraging on the aphid-infested plant, sub-dominant individuals were much more likely to forage off the plants when bean plants were not present. It is likely that changes in the foraging behaviour of sub-dominants in response to plant species identity may underlie the switch in multi-predator effects, possibly due to change in the strength of facilitative interactions. Sub-dominant *Chrysoperla* individuals, in particular, behaved differently depending on whether they were paired with conspecifics or heterospecifics. In the trials where the aphid-free plant was wheat, *Chryspoperla* individuals spent a much higher proportion of time off the plants when paired with *Adalia* than when paired with a conspecific, which may have promoted facilitation between *Adalia* and *Chrysoperla* in the wheat monoculture treatments, mediated by aphid dropping behaviour. However, where the aphid-free plant was bean, foraging allocation to the aphid-free plant by *Chrysoperla* was high and, consequently, their aphid consumption was unlikely to have been affected by aphid dropping behaviour.

Whatever the precise mechanism for the change from positive to negative multi-predator effects on prey suppression as vegetation composition changed from wheat monoculture to wheat and bean polyculture, it is likely that this was mediated by the change of foraging behaviour of individual predators in the presence of extra-floral nectar. Previous studies have shown that the presence of faba bean, compared with the other legumes *Medicago sativa* and *Trifolium pratense*, can cause a marked reduction in aphid consumption resulting in a negative impact of plant species richness on aphid consumption [33]. The effect of faba bean in this earlier study may also have resulted from extra-floral nectar feeding, though this explanation was not proposed by the authors at the time. The impact of plant resources on the functioning of individual natural enemy species are well-documented [34]. Our data suggest that plant resources can also modify the nature of emergent multi-predator effects affecting the efficiency with which multi-predator assemblages suppress prey populations.

There is an open debate about the value of increased natural enemy diversity on pest control in agricultural systems; increasing biodiversity improves pest control in most cases, but in a substantial minority of cases the opposite is true [28], [35], [36]. There is growing evidence that this variability is not only due to intrinsic properties of predators, but may also be influenced by the ecological context. Here we have shown that plant identity, and particularly the provision of plant resources, can also determine the outcome and strength of emergent multi-predator effects by modifying predator behaviour. A caveat to our conclusion is that our experiments were restricted to small temporal and spatial scales and it is not clear that the impact of supplementary resources would be consistent with increasing scale. A short term switch to nectar feeding by *Chrysoperla* may not persist over longer time scales, when feeding on more protein-rich food may be required to maintain growth and development. Similarly, increase in spatial scale would likely reduce encounter rates and the strength of inter-predator interactions. Increase in spatial scale has also been predicted to strengthen richness effects on functioning due to the influence of habitat heterogeneity on species functional complementarity [24], [37], [38], an effect which has been shown to promote positive multi-predator effects among predators [39]. Therefore, we propose that further research at larger temporal and spatial scales is required to fully characterise the interaction between floral resources and the functioning of multi-predator assemblages.
